# Organelle genomes of two *Scaevola* species, *S. taccada* and *S. hainanensis*, provide new insights into evolutionary divergence between *Scaevola* and its related species

**DOI:** 10.3389/fpls.2025.1587750

**Published:** 2025-04-24

**Authors:** Danni Meng, Tianxin Lu, Meng He, Yuze Ren, Mumei Fu, Yuxiao Zhang, Peifeng Yang, Xinyu Lin, Yong Yang, Ying Zhang, Yuchen Yang, Xiang Jin

**Affiliations:** ^1^ Ministry of Education Key Laboratory for Ecology of Tropical Islands, Key Laboratory of Tropical Animal and Plant Ecology of Hainan Province, College of Life Sciences, Hainan Normal University, Haikou, China; ^2^ Hainan Observation and Research Station of Dongzhaigang Mangrove Wetland Ecosystem, Hainan Normal University, Haikou, China; ^3^ Key Laboratory of Xinjiang Phytomedicine Resource and Utilization of Ministry of Education, Key Laboratory of Oasis Town and Mountain-Basin System Ecology of Xinjiang Production and Construction Corps, College of Life Sciences, Shihezi University, Shihezi, China; ^4^ Engineering and Technological Research in Protection and Utilization of Mangrove Rare and Endangered Species, Lingnan Normal University, Zhanjiang, China; ^5^ State Key Laboratory of Biocontrol, School of Ecology, Sun Yat-sen University, Shenzhen, China

**Keywords:** organelle genomes, *Scaevola*, Goodeniaceae, Asteraceae, evolutionary divergence

## Abstract

Chloroplast and mitochondrial genomes harbor crucial information that can be utilized for elucidating plant evolution and environmental adaptation. The organellar genomic characteristics of Goodeniaceae, a sister family to Asteraceae, remain unexplored. Here, using a combination of short-read and long-read sequencing technologies, we successfully assembled the complete organellar genomes of two Goodeniaceae species native to China, *Scaevola taccada* and *S. hainanensis*. Chloroplast genome collinearity analysis revealed that *Scaevola* expanded its genome length through inverted repeat expansion and large single copy fragment duplication, resulting in 181,022 bp (*S. taccada*) and 182,726 bp (*S. hainanensis*), ~30 kb increase compared to its related species. Mitochondrial genomes of two *Scaevola* species exhibit multi-ring topology, forming dual mitochondrial chromosomes of 314,251 bp (*S. taccada*) and 276,175 bp (*S. hainanensis*). Sequence variation analysis demonstrated substantial chloroplast sequence divergence (Pi = 0.45) and an increase in gene copy number within the genus. Relative synonymous codon usage (RSCU) analysis revealed that *Scaevola* chloroplast has a higher bias for A/U-ending codons than mitochondria, with chloroplasts RSCU values ranging from 0.32 to 1.94, whereas mitochondrial RSCU values ranging from 0.38 to 1.62. Phylogenetic analyses support the monophyly of the Asteraceae-Goodeniaceae sister group, whereas the extended evolutionary branches of *Scaevola*, coupled with mitochondrial collinearity analysis, indicate rapid organellar genome evolution of *Scaevola*. Organellar-nuclear horizontal gene transfer analysis identified specific increased in the copy numbers of photosynthesis-related genes and chloroplast-nuclear transfer events in *S. taccada*. Our study not only provides insights for understanding environmental adaptation mechanisms of coastal plants, but also contributes to elucidating organellar genome evolution in *Scaevola* and Goodeniaceae.

## 1 Introduction

Goodeniaceae is a distinctive taxon within the Asterales order of angiosperms, comprising approximately 11 genera and over 400 species, primarily originating from the Australian continent, where about 95% of species remain endemic (Ghisalberti, 2004). Molecular phylogenetic studies suggest that Goodeniaceae shares a common ancestor with Asteraceae, with their divergence occurring approximately 80 million years ago, highly consistent with the geological timing of Australian continent’s separation from the Gondwana supercontinent (Shen et al., 2023). *Scaevola* is the only genus within Goodeniaceae that has successfully dispersed beyond Australia, expanding into tropical and subtropical coastal zones through efficient long-distance dispersal mechanisms, demonstrating its remarkable propagation capability and environmental adaptability (Jabaily et al., 2014). *S. taccada* is widely distributed across the coastlines of Pacific islands and Indian Ocean, and has spread to the Caribbean region as an invasive species, potentially leading to ecological threats to native species in Puerto Rico, such as *S. plumieri* (Swensen et al., 2024). As a typical coastal pioneer plant, *S. taccada* exhibits high tolerance to salt spray, drought resistance, and wind-breaking, as well as the capability for sand-stabilizing (Lee et al., 2020; Starman and Lombardini, 2006; Toscano et al., 2020). These advantages make it an ideal plant resources for coastal ecosystem restoration (Walker et al., 1997). Extracts from *S. taccada* exhibit antiviral and anticancer activities, suggesting potential medicinal value (Locher et al., 1996). Population genetic studies have uncovered complex gene flow patterns and substantial population differentiation in *S. taccada*, shedding light on the adaptive evolution and dispersal mechanisms of coastal species. In the Hawaiian Islands, *Scaevola* exhibits a typical case of hybrid speciation (Howarth and Baum, 2005). However, the limited molecular characterization of *Scaevola* species contrasts with their significant ecological and potential medical values.

Plant organelle genomes are indispensable for elucidating plant origin, evolution, and adaptation, due to their unique genetic traits, including maternal inheritance, high conservation, and abundant copy numbers, as well as their critical physiological functions in photosynthesis and energy metabolism (Wang et al., 2024a). The chloroplast genome is structurally conserved, typically containing 110-130 genes, with only 0.5-1.0 base substitutions per million years, making it an ideal molecular marker for reconstructing higher-order phylogenies (Curtis and Clegg, 1984). For instance, comparative analysis of Asterales chloroplast genomes has revealed early divergence events between Goodeniaceae and Calyceraceae (Panero and Crozier, 2016). Additionally, simple sequence repeat (SSR) markers developed from chloroplast intergenic regions, such as trnL–*trnF* and *psbA–trnH*, have been successfully applied in genetic diversity studies of crop wild relatives (Lima et al., 2021; He et al., 2024). Emerging research has demonstrated that chloroplast genomes in halophytes often display unique selection patterns and retrograde signalings. For instance, adaptive evolution of the *ndhF* gene may play an important role in resistance against high-salt environments (Zheng et al., 2025), while the expression of some salt-tolerance genes is also influenced by organelle genomes (Robles and Quesada, 2019). In contrast, mitochondrial genomes have greater dynamic complexity, including horizontal gene transfer, frequent recombination, and substantial size variation (0.1–10 Mb), which are closely linked to plant regulatory mechanisms for environmental adaptation (Alverson et al., 2010). Mitochondrial genes, such as *cox1*, *nad5*, and *atp6*, exhibit significant positive selection signals under environmental stress (Qiu et al., 2021). However, despite the NCBI database has cataloged approximately 13,000 plant chloroplast genomes, these records predominantly focus on economic crops and model plants, with research on wild plants remaining insufficient. Compared to chloroplast, the number of published plant complete mitochondrial genomes (~673) is one to two orders of magnitude lower (Wang et al., 2024b). The application of long-read sequencing technologies has significantly advanced organelle genome research, especially in resolving complex structures and repetitive sequences in mitogenomes (Liu et al., 2024).

In China, only two *Scaevola* species are distributed: the widespread *S. taccada* and the China-Vietnam endemic species *S. hainanensis* (Flora of China, 2020). Chromosome-level genome assembly reveals that, compared to *S. hainanensis*, *S. taccada* has undergone specific gene family expansions and adaptive modifications to withstand coastal environmental stresses, such as intense light, high salinity air, and poor soil conditions in coastal areas (Li et al., 2023). Within the Goodeniaceae family, only one chloroplast genome sequences of *Scaevola* has been reported, as outgroup to a large-scale study of the Caryophyllales (Yao et al., 2019). Population genetic study based on chloroplast SSR markers of *S. taccada* has revealed its dispersal pathways of Western Pacific island populations (Banerjee et al., 2022). However, mitochondrial genome studies in *Scaevola* remains largely unexplored, limiting our comprehensive understanding of its evolutionary diversity.

In this study, we employed short-read and long-read sequencing technologies to assemble the organellar genomes of two *Scaevola* species native to China. We conducted comparative analyses of the organellar genomes between these two species, including gene contents, repeat distributions, codon usage, genomic structural variations, RNA editing sites and phylogenetic relationships. An in-depth investigation of *Scaevola* organellar genomes not only contributes to elucidating nuclear-organellar genome co-evolution, but also provides critical insights for understanding the evolutionary divergence between *Scaevola* and its related species.

## 2 Materials and methods

### 2.1 Plant material and DNA sequencing

Young leaves of *S. taccada* and *S. hainanensis* were harvest from individuals planted in the plant garden of the college of life sciences in Hainan Normal University. Leaves were immediately frozen in liquid nitrogen and ground into fine powder. Genomic DNA extraction was performed using the CTAB method. DNA quality and quantity were evaluated using NanoDrop (Thermo Fisher Scientific, USA). Sequencing was carried out on the Illumina NovaSeq 6000 (Illumina Inc., USA) to generate paired-end short reads (2 × 150 bp). The Oxford Nanopore PromethION platform (Oxford Nanopore Technologies, UK) was employed to generate long-reads. High-quality short reads were generated by adapter trimming and quality filtering using fastp software, while long reads were used to facilitate the assembly of the complex structural architecture of organellar genomes.

### 2.2 Organellar genomes assembly

The chloroplast genome was assembled using ptGAUL with the parameters -t 20 -f 3000 (Zhou et al., 2022). The long-read Nanopore data were used for initial contig construction. Illumina short reads were mapped to the draft assembly using BWA v0.7.17 (Li and Durbin, 2009) for base error correction. Assembly errors were corrected through multiple rounds of iterative polishing using Pilon. v1.24 (Walker et al., 2014).

The mitochondrial genomes were assembled using GSAT v1.1.2 (He et al., 2023a), a hybrid assembler that integrates Illumina and Nanopore data. Flye v2.9 (Kolmogorov et al., 2019) was employed as an auxiliary assembly tool to ensure the comprehensiveness of the long-read assembly. Assembly graphs were visualized and validated using Bandage v0.8.1 (Wick et al., 2015). Conflicting regions between assemblies were resolved by cross-verification, and complex structural variations (e.g., repeats or rearrangements) were validated by BLASTN alignment (-task megablast) of Nanopore reads (Camacho et al., 2009). Only structures supported by long-read coverage over 10× were retained.

### 2.3 Genome annotation and visualization

Chloroplast and mitochondrial genomes were annotated using GeSeq (Tillich et al., 2017) with default parameters. Transfer RNA and ribosomal RNA genes were manually curated by comparison with closely related species. Open reading frames (ORFs) were predicted using ORFfinder (NCBI). The annotated genomes were visualized with OGDRAW (Greiner et al., 2019).

### 2.4 Sequence features analyses

Simple sequence repeats (SSR) were identified using MISA (http://pgrc.ipk-gatersleben.de/misa/) with the minimum repeat parameters set to 10, 6, 4, 3, 3, and 3 repeats for mono-, di-, tri-, tetra-, penta-, and hexa- nucleotide SSRs, respectively. The online software REPuter (https://bibiserv.cebitec.uni-bielefeld.de/reputer) was used to detect repeat types and numbers for long sequence repeats, with a maximum computed repeats of 50 and minimal repeat size of 30  bp. Potential RNA editing sites in protein-coding genes were predicted using PREP Suite online tools (http://prep.unl.edu/) with default parameters. MEGA10 was used to calculate relative synonymous codon usage (RSCU) values to quantify the codon usage patterns. And DNaSP5 component of MEGA10 was used to calculate synonymous (Ks) and nonsynonymous (Ka) substitution rates for evaluating selective pressure.

### 2.5 Synteny and nuclear-organellar DNA transfer analyses

Syntenic relationships between *Scaevola* and its close relatives were analyzed using SyRI v1.6.3 (Goel et al., 2019), with visualization performed via plotsR. Potential nuclear-cytoplasmic gene transfers were identified by BLASTN alignment of organellar genomes against the nuclear genome, using the following parameters: -word_size 9 -evalue 1e-5 -reward 2 -gapopen 5 -gapextend 2 -penalty -3. Regions with >75% identity and length >100 bp were retained (Richardson and Palmer, 2007). Results were visualized using NGenomeSyn (He et al., 2023b).

### 2.6 Phylogenetic analysis

Maximum likelihood phylogenetic trees were reconstructed using IQ-TREE V.1.6.8 in PhyloSuite v1.2.3 (Zhang et al., 2020) under the Partition Mode, with 1000 bootstrap replicates. Alignments were performed using the chloroplast and mitochondrial protein-coding genes from *Scaevola* and related taxa, respectively. Phylogenetic trees were visualized and annotated using iTOL (Letunic and Bork, 2021).

## 3 Results

### 3.1 Assembly and sequence features of the organellar genomes of two *Scaevola* species

**Figure 1:**
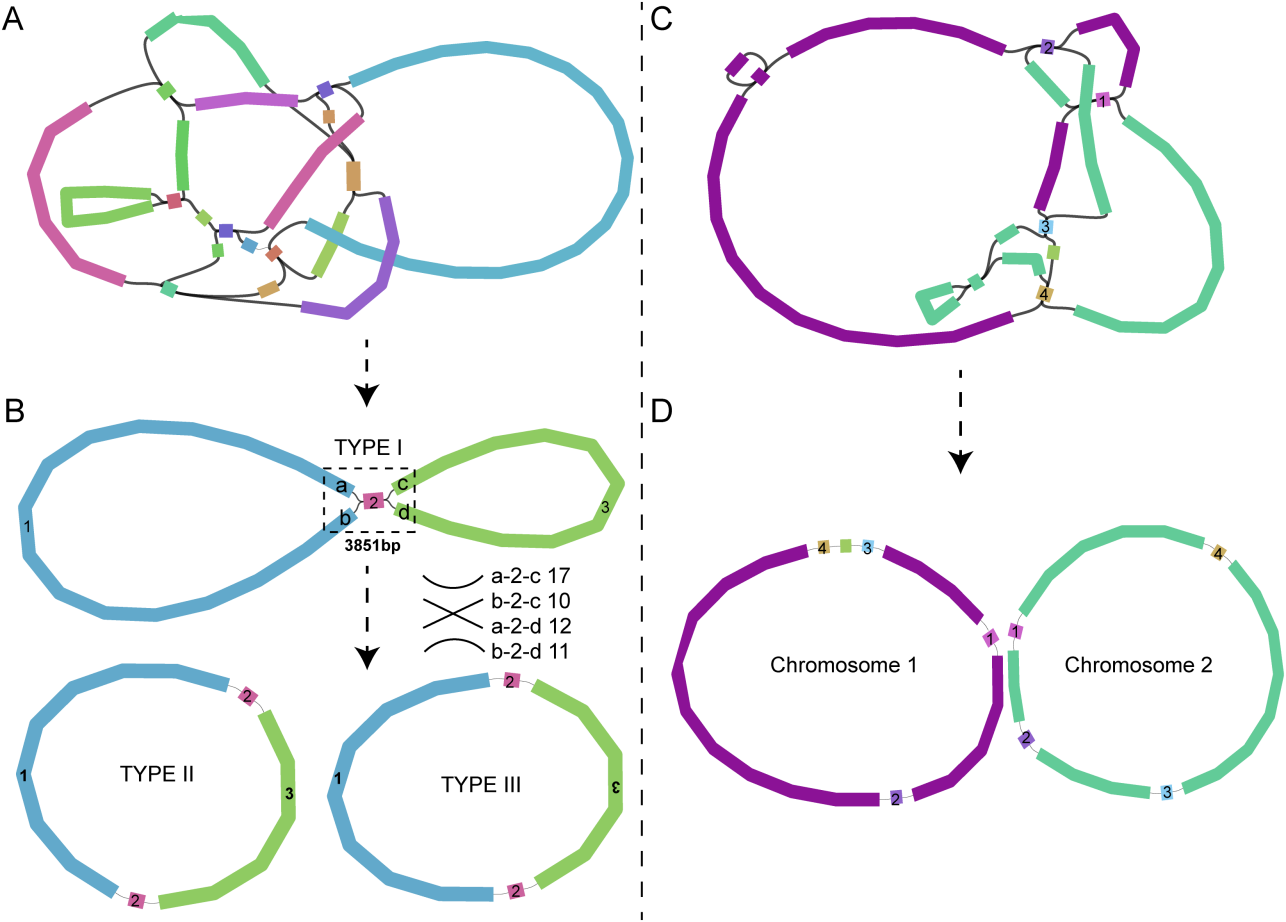
Mitochondrial genome assemblies of *S. taccada* **(A, B)** and *S. hainanensis* **(C, D)**. The draft mitochondrial genomes **(A, C)** were assembled using Illumina short-reads data, and the final chromosomes **(B, D)** were further determined using long-reads data. In each of the panel **(A, C)**, the scaffolds connected together (on the same long read) are presented by the same colors.

Sequencing of DNA extracted from leaves of two *Scaevola* species yielded 16 Gb, 15 Gb Illumina short-reads and 26.6 Gb and 10.3 Gb Nanopore long-reads for *S. taccada* and *S. hainanensis*, respectively. Multi-circular topological structures were generated for the mitochondrial genome assemblies (**Figure 1**). Numerous studies have reported the existence of multi-circular molecules in plant mitochondria (Butenko et al., 2024; Wang et al., 2025; Wu et al., 2022). Consequently, manual corrections were performed by aligning long-reads data to shared fragments that could potentially exhibit multiple connection patterns. For *S. taccada*, one shared fragment was identified as a forward repeat, with all possible scaffold arrangements supported by the long-reads. This led to the assembly of three potential mitochondrial genome topological structures (TYPE I, II and III) sharing identical scaffold sequences but different arrangements (**Figure 1B**). These alternative topological structures affected only the scaffold connection orientations without altering gene content, therefore, they were considered as a single circular chromosome in subsequent analyses. However, for *S. hainanensis*, two chromosomes with different sequences (resulting from distinct topological structures) were finally identified (**Figure 1D**) and analyzed as separate chromosomes (chromosome1 and chromosome2) in subsequent analyses.

The statistical information of chloroplast and mitochondrial genomes of two *Scaevola* species is provide in **Tables 1**, **2**. Generally, the chloroplast genomes of *S. taccada* and *S. hainanensis* are 181,022 bp and 182,726 bp in length, which are longer than the typical chloroplast genome of angiosperm (115–165 kb). Accordingly, the protein coding gene numbers are 88 and 86 for *S. taccada* and *S. hainanensis* (**Table 1**). The mitochondrial genomes of *S. taccada* and *S. hainanensis* are 314,251 bp and 276,175 bp in length, both encoding 24 proteins (**Table 2**). Detailed gene names and gene classifications of *S. taccada* and *S. hainanensis* are shown in **Supplementary Tables S1**, **S2**.

**Table 1:** Comparison of chloroplast genome features of two *Scaevola* species.

Feature		*Scaevola taccada*	*Scaevola hainanensis*
Genome size (bp)		181,022	182,726
LSC (bp)		98,455	95,830
SSC (bp)		8,563	8,502
IRs (bp)		37,002	39,197
Protein coding genes		88	86
Number of rRNA genes		4	4
Number of tRNA genes		42	43
G+C %	LSC	36	36
	SSC	31	31
	IR	38	38
	Total genome	37	37

**Table 2:** The mitochondrial genome data of two *Scaevola* species.

Feature	*Scaevola taccada*	*Scaevola hainanensis*
Structure	Circular	Circular
Circular molecular number	1	2
Genome size (bp)	314,251	276,175
G+C %	44.36	44.56
Protein codon genes	24	24
Number of rRNA genes	3	3
Number of tRNA genes	13 (3)	13 (3)

**Figure 2:**
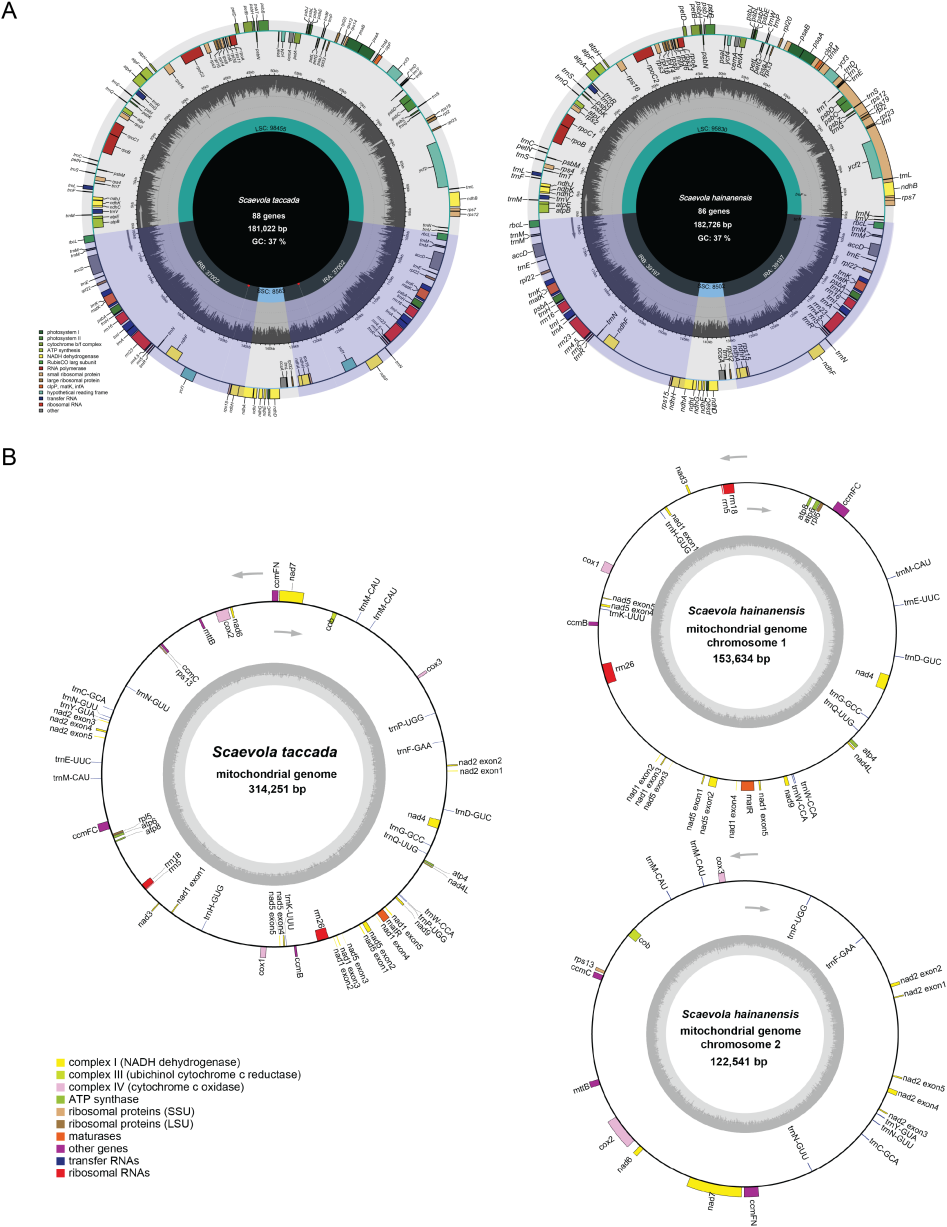
Circular maps of the chloroplast **(A)** and mitochondrial **(B)** genomes of *S. taccada* and *S. hainanensis*. For each panel, genes outside the circles are transcribed clockwise and genes inside the circle are transcribed counter-clockwise. The dark-gray inner circle represents GC contents and light-gray one represents the AT contents. The coding genes belonging to different classes are presented by different colors.

**Figure 3:**
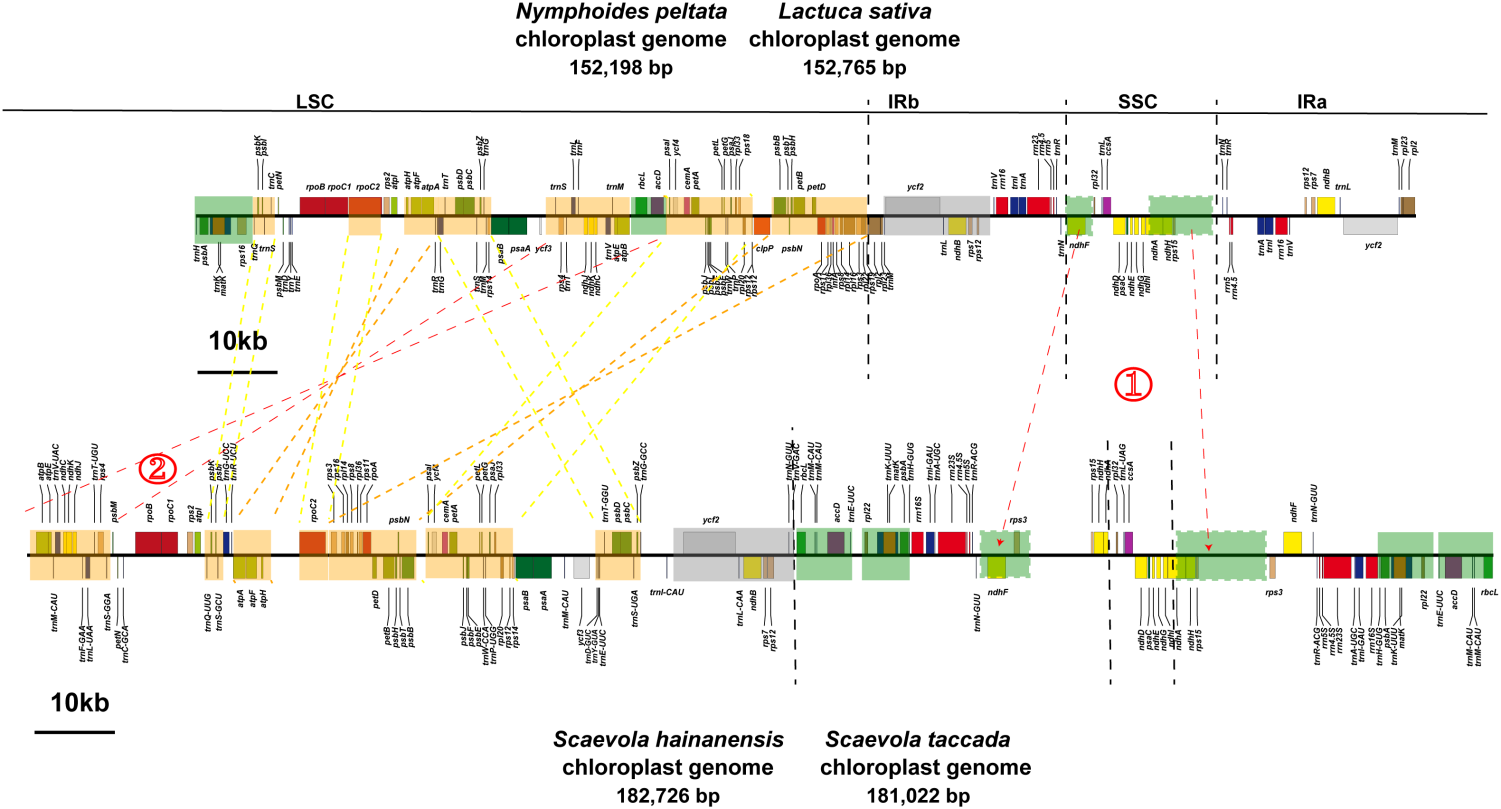
Fragment rearrangement in the LSC region and IR expansion in the chloroplast genomes of two *Scaevola* species. The orange blocks represent fragment rearrangement in the LSC region, while the green blocks represent IR expansion and SSC contraction.

The circular maps of organellar genomes of two *Scaevola* species were conducted to illustrate their structure features. The plastomes are consist of the typically conserved four distinctive parts (**Figure 2A**). The lengths of chloroplast genome of *Scaevola* are around 30 kb longer than the typical chloroplast genome of the family Asteraceae, which shares a common ancestor with the family Goodeniaceae. Notably, both *Scaevola* species have a short single copy (SSC) of 8.5 kb, much shorter than that of Asteraceae species, which are already reported as “small SSC” (Cho et al., 2024; Chen et al., 2022). To investigate the reason for this phenomena, we performed collinear analysis between *Scaevola* plastomes and two Asterales plastomes (*Nymphoides peltata* from Menyanthaceae and *Lactuca sativa* from Asteraceae). Results showed that a fragment duplication and rearrangement events in LSC, as well as a duplication event in SSC, together leading to IR expansion and SSC contraction in *Scaevola* species (**Figure 3**). The LSC duplication and rearrangement events included some protein coding regions that are important for photosynthesis, resulting in gene duplication in chloroplast genomes of *Scaevola*, such as *rbcL*. The copy numbers of several important chloroplast coding genes are also duplicated in *Scaevola* compared to 13 related species, including *accD*, *matK*, *ndhF*, *ndhH*, *psbA*, *rbcL*, *rpl22* and *rps15* (**Supplementary Figure S1**).

### 3.2 Sequence variation and codon usage of organellar genomes of two *Scaevola* species

The chloroplast genomes of *S. taccada* and *S. hainanensis* are conserved in general (identity of 98.17%), with several regions showing substantially high variation. As is shown, the nucleotide diversity (Pi) of the two chloroplast genomes is higher than 0.1 in the genome regions of *rpoC2-rps3*, *rps3-rpl16*, *trnM-ycf3*, *rpl2-rpl23* (with the highest Pi value of 0.45), *ycf2*, *accD-trnE*, and *ycf1* (**Supplementary Figure S2**).

**Figure 4:**
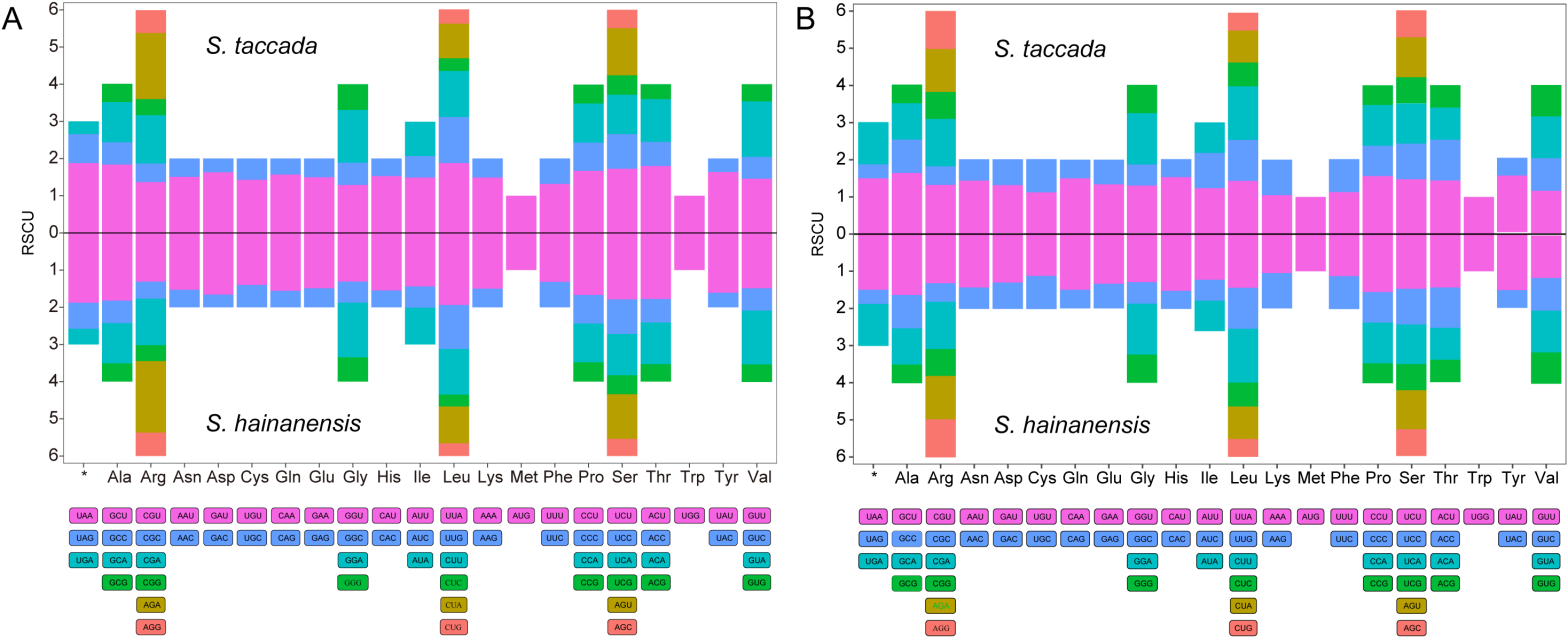
Relative synonymous codon usage (RSCU) histogram of the chloroplast **(A)** and mitochondrial **(B)** genomes of two *Scaevola* species. For each amino acid, different codons are shown in different colors.

RSCU of organellar genomes of both *Scaevola* species were analyzed. A total of 24,050 and 25,742 codons were identified in the protein coding genes (PCGs) of chloroplast genomes of *S. taccada* and *S. hainanensis* (**Supplementary Table S3**), as well as 7,685 and 7,988 codons in their mitochondrial genomes (**Supplementary Table S4**). Leucine is the most frequently used amino acid (11.26%), followed by isoleucine (8.45%) and serine (7.81%), while cysteine has the lowest abundance, with a proportion of 1.17%. Consistent with previous studies, organellar PCGs of *Scaevola* showed strong bias on A/U-ending codons, with RSCU values > 1 (**Figure 4**). The RSCU values of all chloroplast codons ranged from 0.32 (CUC for leucine in *S. hainanensis*) to 1.94 (UUA for leucine in *S. hainanensis*). For mitochondrial genome, the RSCU values ranged from 0.38 (UAG for stop codon) to 1.62 (GCU for alanine in *S. hainanensis*).

**Figure 5:**
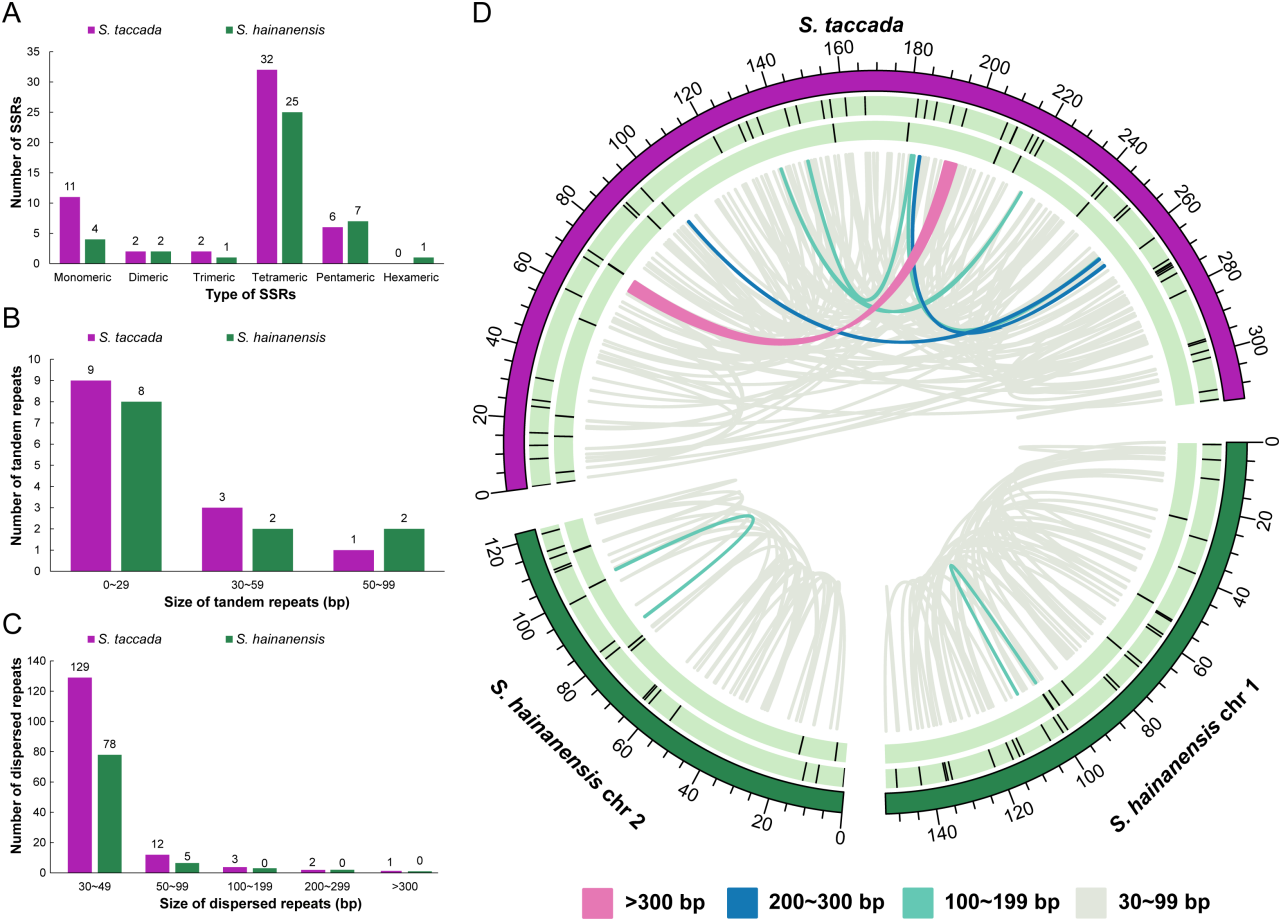
The distribution of repeat elements in two mitochondrial genomes. **(A)** The frequency of identified SSRs. **(B)** The frequency of identified tandem repeats. **(C)** The frequency of identified dispersed repeats. **(D)** Distribution of repeat elements in mitochondrial genomes. The outer circle represents the SSRs, followed by tandem repeats and the inner lines represent the dispersed repeats.

Furthermore, repeat sequences in the organellar genomes of two *Scaevola* species were analyzed. In the mitochondrial genomes, the most abundant SSR is tetrameric repeats (32 and 25 in *S. taccada* and *S. hainanensis*). The palindromic dispersed repeats are more in *S. hainanensis* (80) than in *S. taccada* (42), while the longest palindromic repeats in *S. taccada* (3,871 bp) is much longer than in *S. hainanensis* (169 bp) (**Supplementary Table S5**; **Figure 5**).

A total of 289 and 287 potential RNA editing sites were identified in chloroplast genomes of *S. taccada* and *S. hainanensis*, with the *ndhB* gene possessing the most RNA editing sites (22 and 19 in *S. taccada* and *S. hainanensis*). Additionally, 347 and 350 RNA editing sites were identified in the mitochondrial genomes of two *Scaevola* species, with the *ccmB* gene possessing the most RNA editing sites (32 and 33 in *S. taccada* and *S. hainanensis*) (**Supplementary Figure S3**).

### 3.3 Phylogenetic and syntenic analyses of organellar genomes of two *Scaevola* species

**Figure 6:**
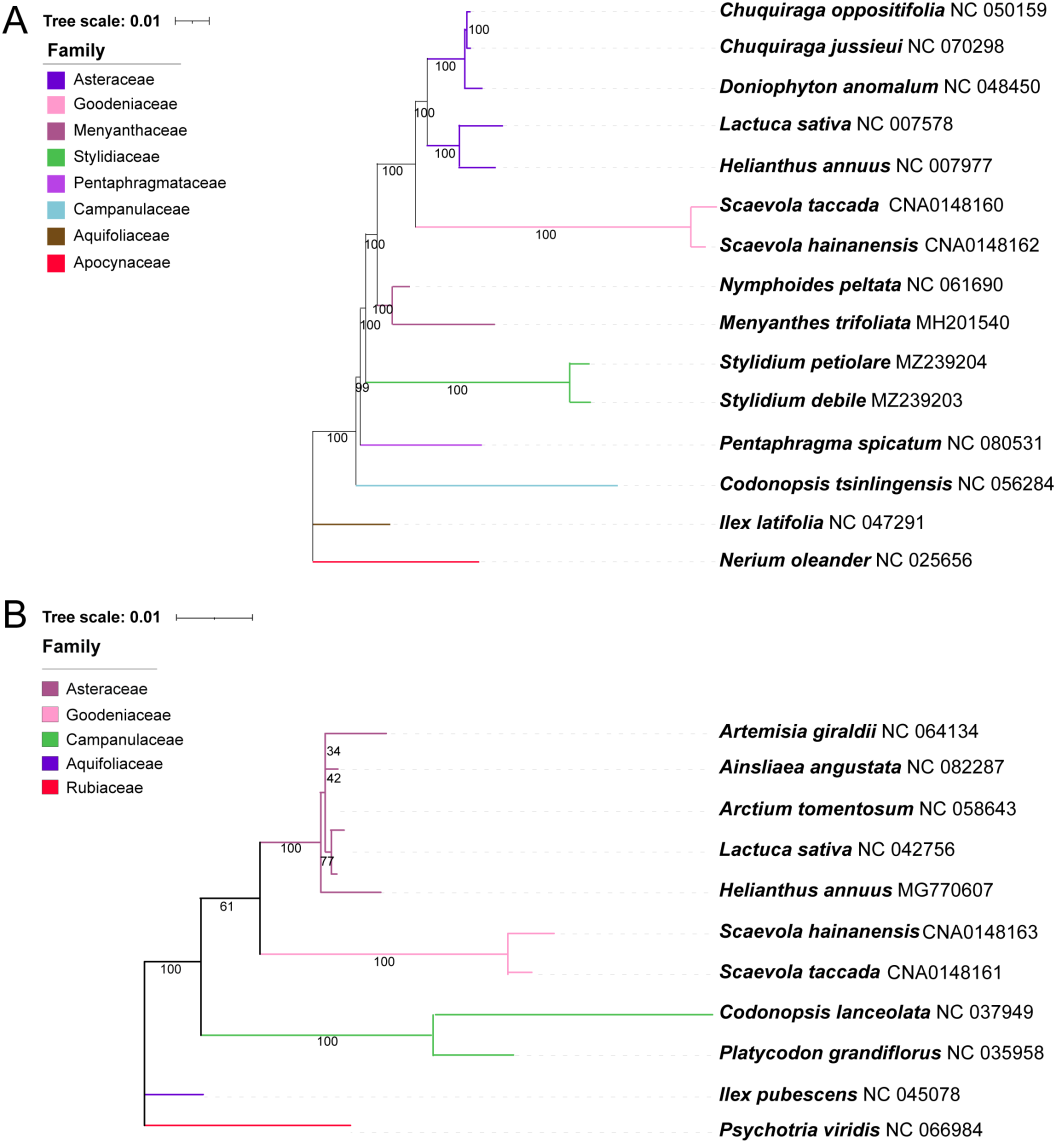
Phylogenetic tree based on 62 common chloroplast genes **(A)** and 17 conserved protein coding genes of mitochondria **(B)** using the IQtree. Bootstrap values obtained from 1000 replicates are shown below the clades. Different families are shown in different colors. Nerium oleander and *Ilex latifolia* were used as outgroups for phylogenetic tree of the chloroplast genes and *Ilex pubescens* and *Psychotria viridis* for mitochondria genes.

Phylogenetic trees were constructed using the chloroplast and mitochondrial genomes of *S. taccada* and *S. hainanensis*, and other related species, respectively. Complete chloroplast and mitochondrial genomes of Goodeniaceae are limited to only two species reported in this study. The chloroplast genomes of 5 representative Asteraceae species and 6 Asterales species other than Asteraceae were obtained from NCBI Genbank. In the tree constructed using 62 common chloroplast genes, the two *Scaevola* species formed a clade and exhibited the closest relationship with Asteraceae species (**Figure 6A**). For mitochondrial genomes, much fewer sequences are available, leading to a smaller scale of genome dataset for phylogenetic analysis. Seventeen common mitochondrial genes were used to construct a phylogenetic tree of 11 species, including 7 representative Asterales species, 2 *Scaevola* species, and 2 outgroups (*Ilex pubescens and Psychotria viridis*). The branches of mitochondrial based phylogenetic trees are longer than those of chloroplast genome based trees, indicating a faster nucleotide substitution rate in mitochondrial genome (**Figure 6B**). A bigger tree performed with 50 available mitochondrial genomes of Asterales species showed similar branch length (**Supplementary Figure S4**). The selective pressure analysis further revealed higher Ka/Ks values in mitochondrial-coded genes compared to chloroplast-coded genes (**Supplementary Figure S5**), indicating that the organelle evolutionary strategies of *Scaevola* species may be influenced by their environmental pressures, especially concerning the mitochondrial genome.

**Figure 7:**
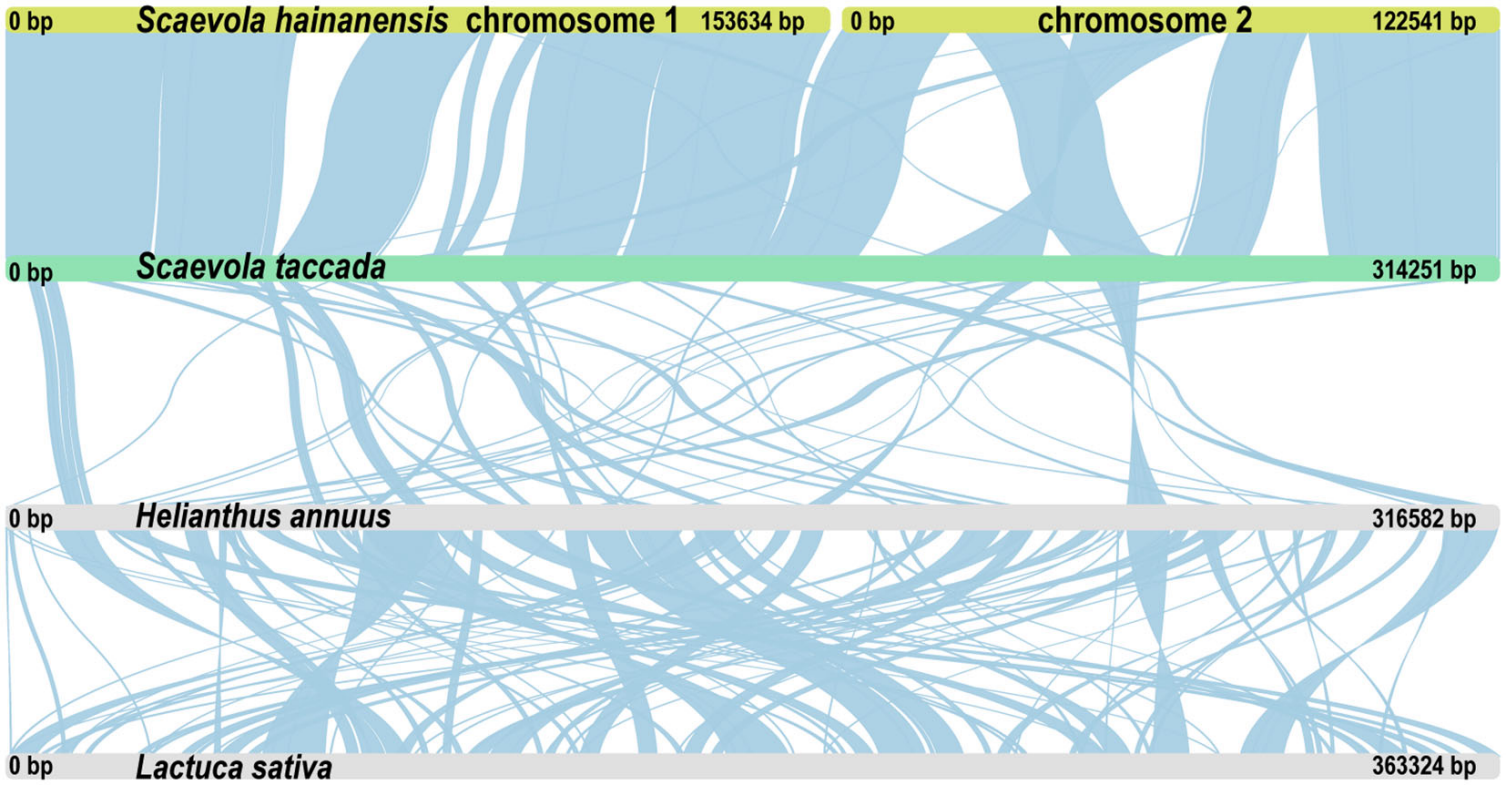
Whole mitogenome collinearity analysis among *S. taccada*, *S. hainanensis*, *Helianthus annuus* and *Lactuca sativa*. Long strips of different colors represent different plant mitochondrial genomes. Liner blocks represent collinear segments.

**Figure 8:**
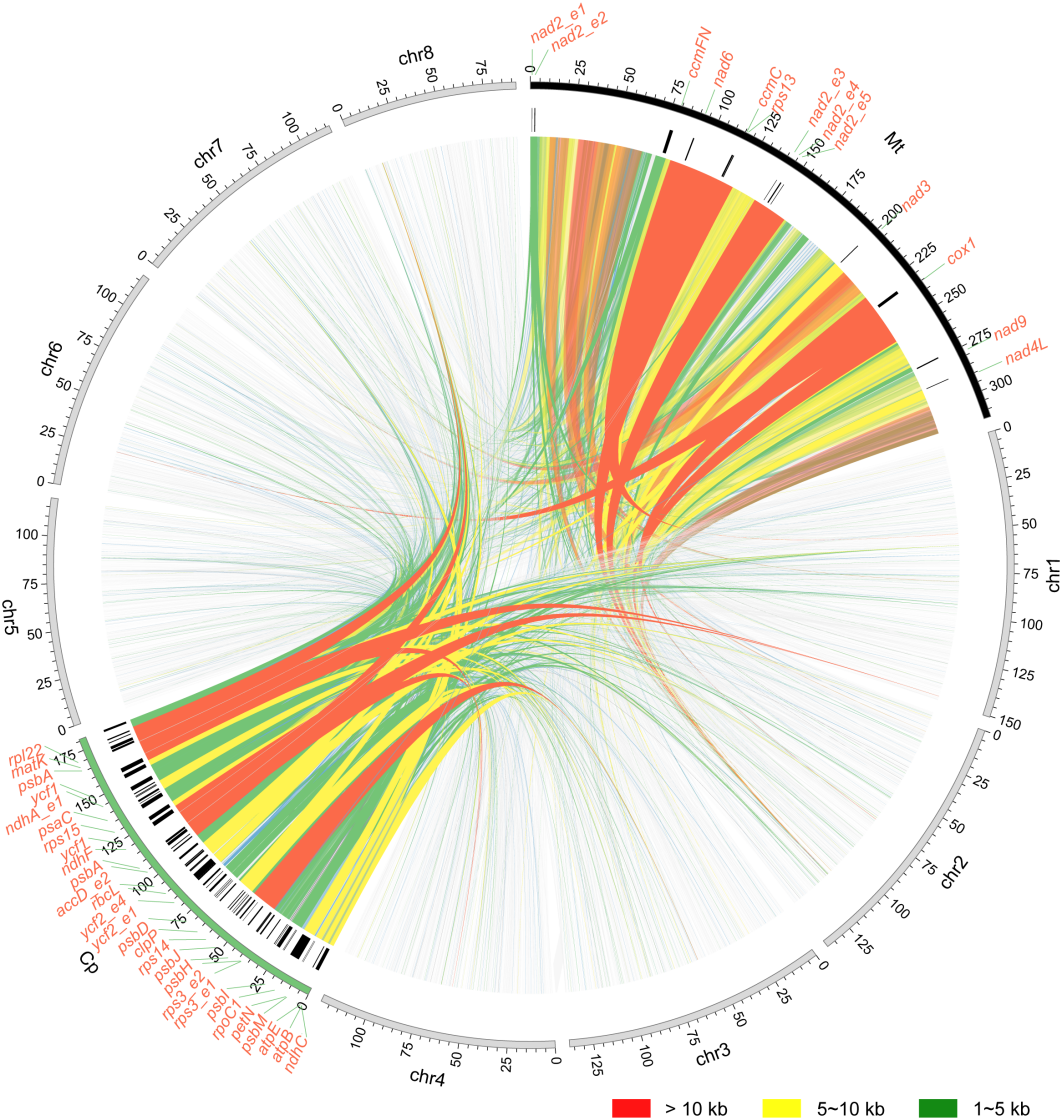
Schematic representations of mitochondrial plastid DNA (MTPT) and nuclear–mitochondrial DNA segments (NUMT) of S. *taccada.* Collinear segments of different length are presented by different colors: red, >10 kb; yellow, 5–10 kb; green, 1–5 kb. Names of the genes within the collinear segments are shown at the corresponding positions.

Syntenic analysis was performed to investigated the mitochondrial genome sequences and structure variations (**Figure 7**). The mitochondrial genomes of two *Scaevola* species exhibited high collinearity. However, compared to representative species of related family Asteraceae, only a few collinear segments could be identified, with a large portion of rearrangement, indicating fast evolutionary divergence of mitogenome between *Scaevola* and its related species. Further, to investigate the horizontal gene transfer between organellar genomes and the nuclear genome, syntenic fragments were also identified by comparing organellar genomes to each other and to nuclear genome (**Supplementary Figure S6**). Notably, *S. taccada* showed substantially more syntenic segments over 10 kb between the mitochondrial genome and the nuclear genome. Further examining these fragments in detail helped identify horizontal gene transfer in *S. taccada*. PCGs within the syntenic segments were labeled outside the circle in **Figure 8**. Some proteins important for photosynthesis were transferred to the nuclear genome, including *rbcL*, *psaC*, *psbA*, and *psbH*, while some important mitochondrial coding genes were also transferred to the nuclear genome, including *ndh6* and subunits of *nad2* complex.

## 4 Discussion

Plants of the *Scaevola* have important ecological value as pioneer species on tropical coral islands. They also possess high ornamental value, characterized by their symmetrical fan-shaped colorful flowers, so called “fan flowers”. Some species have been introduced to countries outside Australia as horticultural plants. The lack of genomic data has been a major limiting factor in molecular evolution research on *Scaevola*, despite previous studies developing several markers to investigate the phylogeny of this genus (Ando et al., 2014; Emura et al., 2022). Goodeniaceae shares the most recent common ancestor with Asteraceae, considered as the “most advanced” family of angiosperms. After diverging from Asteraceae approximately 80 million years ago, Goodeniaceae underwent a radiation of approximately 400 species across the Australian continent (Ghisalberti, 2004). However, the complete chloroplast genome of only one species of Goodeniaceae (*S. taccada*) has been reported in a large-scale study of the Caryophyllales (Yao et al., 2019). This study presents the first complete organellar genomes (chloroplast and mitochondrial) for two Goodeniaceae species. The chloroplast genome of *Scaevola* is larger than that of representative Asteraceae plants but possesses a very small SSC (**Table 1**; **Figure 2**). Our study reveals that this phenomenon is resulted from duplication and rearrangement of LSC fragments, as well as duplication of SSC fragments, causing an IR expansion of approximately 10 kb each (**Figure 3**). This LSC duplication and IR expansion led to increased copy numbers of some chloroplast encoded genes, including several photosystem II complex members and *rbcL* gene (**Supplementary Table S1**; **Supplementary Figure S1**), which may correlate with the adaptation of *Scaevola* plants to tropical island habitats characterized by high light intensity and drought conditions. Expanding sampling across Goodeniaceae will be essential to elucidate the evolutionary significance and ecological implications of this organellar genomic architecture.

Compared to chloroplast genomes, plant mitochondrial genomes exhibit greater variation and a broader range of lengths, from 66 kb in *Viscum scurruloideum* to 12 Mb in *Larix sibirica* (Wu et al., 2022; Putintseva et al., 2020). The widespread replication events and repetitive fragments in mitogenomes significantly increase the difficulty of their assembly, often resulting in complicated topological multiple circular molecules (Wang et al., 2024a). Long-reads sequencing technologies greatly improve assembly challenges. Benefit from the application of Nanopore long-read sequencing, we simplified the complex topological structure of *S. taccada* mitochondrial genome into two large circles and one shared fragment (3,871 bp), ultimately obtaining circular molecules with identical sequences but two different connection patterns. While the topological structure of *S. hainanensis* mitochondrial genome was more complex, finally resolved into two distinct circular molecules with different sequences (sharing four short repetitive sequences), defined as chromosome 1 and chromosome 2 (**Figure 1**). The current disparity between sequenced mitochondrial (~600) and chloroplast (~13,000 NCBI entries till September 2023) genomes will be diminished with the aid of long-read sequencing technologies.

In this study, the GC contents of *Scaevola* mitochondrial and chloroplast genomes were 44% and 37%, similar to those reported Asteraceae plants (**Tables 1**, **2**). The mitochondrial genome sizes of the two *Scaevola* species were 314,251 bp (*S. taccada*) and 276,175 bp (*S. hainanensis*), smaller than most reported Asteraceae plants and containing fewer protein-coding genes, while the chloroplast genomes were larger and contained more protein-coding genes (Wang et al., 2024b). These different organelle genome evolutionary patterns between *Scaevola* and their Asteraceae related species may be related to their survival strategies and adaptations to different habitats. RSCU analysis revealed that *Scaevola* chloroplast has a higher bias for A/U-ending codons than mitochondria, with many A/U-ending codons in chloroplasts having RSCU values exceeding 1.5 (maximum 1.94), while fewer mitochondrial codons had RSCU values exceeding 1.5 (maximum 1.62) (**Figure 4**; **Supplementary Tables S3**, **S4**). This lower preference for A/U-ending codon may contribute to the higher GC content observed in mitochondrial genomes. Compared to chloroplasts, mitochondria endure greater oxidative stress and therefore face stronger pressure to maintain genomic stability, typically resulting in higher RNA editing sites and efficiency (Hu et al., 2024). In both *Scaevola* species reported in this study, over 70% of mitochondrial genes possess more than 10 RNA editing sites (maximum 33), while most chloroplast genes have fewer than 5 predicted RNA editing sites (maximum 22), which is consistent with current understanding (**Supplementary Figure S3**).

Within the order Asterales, the phylogenetic position of Goodeniaceae is relatively well established, with current evidence strongly supporting its sister-group relationship to Asteraceae, as demonstrated by the phylogenetic analyses in this study based on both chloroplast and mitochondrial genomes (**Figure 6**; **Supplementary Figure S4**). However, due to the lack of organellar genome information, this study was unable to resolve the currently controversial phylogenetic relationships among different genera within the family Goodeniaceae. In the current era of big data, sampling and sequencing more organelle genomes of representative Goodeniaceae species could help address this challenge. Nevertheless, we noticed that compared to other well-studied genera, such as genus *Saussurea* in Asteraceae (Mahai et al., 2024), the chloroplast genomes within genus *Scaevola* exhibited a higher level of sequence divergence, with the highest Pi value reaching 0.45 (**Supplementary Figure S2**), whereas many chloroplast genome studies show Pi values less than 0.1 (Fang et al., 2024; Jin et al., 2023). Both chloroplast and mitochondrial genome-based phylogenies exhibited longer evolutionary branch lengths for *Scaevola* (**Figure 6**; **Supplementary Figure S4**), indicating high levels of sequence divergence between Goodeniaceae and Asteraceae, within Goodeniaceae, and even within the genus *Scaevola*. This reflects a faster rate of nucleotide substitution, suggesting that Goodeniaceae may be undergoing rapid radiation, potentially linked to the relatively recent emergence of Asteraceae and Goodeniaceae in the evolutionary history of angiosperms (Zhang et al., 2024). Mitochondrial genome collinearity analysis also revealed strong collinearity between *Scaevola* plants, with several large fragment insertions and inversions, whereas between *Scaevola* and related Asteraceae plants, almost no large collinearity blocks were found in their mitochondrial genomes (**Figure 7**). This indicates that Goodeniaceae and Asteraceae plants have experienced substantial structural variation and rapid mitochondrial genome evolution since their divergence 80 million years ago. Notably, in *S. taccada*, one of the few Pacific-Indian Ocean widespread species in Goodeniaceae, we observed not only chloroplast genome fragment duplication events leading to increased copy numbers of important photosynthetic genes (such as *rbcL*, falling into the IR region), but also horizontal transfer of these genes between chloroplast and nuclear genomes (such as *rbcL*, **Figure 8**). These phenomena were not observed in the regionally restricted species *S. hainanensis*, which may be related to *S. taccada*’s extensive adaptation to the high light intensity, low water retention, high air salinity, and nutrient-poor soil environments of tropical coral islands across the Pacific-Indian Ocean (Li et al., 2023).

## 5 Conclusions

This study presents the first group of organellar genomes from *Scaevola* plants, revealing their unique evolutionary patterns. The chloroplast genomes of *Scaevola* plants have increased in length through IR expansion and LSC duplication, making them approximately 30 kb longer than those of their sister group Asteraceae. Meanwhile, the mitochondrial genomes exhibit multi-circular topological structures (dual-chromosome model). Significantly accelerated variation (maximum Pi = 0.45) and gene copy number expansion were observed in both *Scaevola* species, whereas their mitochondrial genomes showed size reduction and increased RNA editing sites, suggesting their divergent evolutionary strategies. Phylogenetic analyses confirm the monophyly of the Asteraceae-Goodeniaceae group, yet the remarkable divergences in organellar genome structures between these families suggest independent evolutionary paths. The adaptive expansion of chloroplast genomes (such as increased *rbcL* copy numbers) and horizontal gene transfer events (chloroplast-to-nuclear genome) may be closely associated with the adaptive radiation of the widespread of *S. taccada* to tropical coral island habitats, characterized by intense light, high air salinity and drought conditions. These findings provide organelle-level insights into the evolutionary divergence between *Scaevola* plants and their related species.

## Data Availability

The complete organellar genome sequences generated in this study have been deposited in the China National GeneBank DataBase (CNGBdb; https://db.cngb.org/) under accession numbers CNA0148160, CNA0148161, CNA0148162, and CNA0148163. These annotations are publicly accessible through the CNGBdb repository search interface. Further inquiries can be directed to the corresponding author.
