# Supplementary material for: Organelle genomes of two Scaevola species, S. taccada and S. hainanensis, provide new insights into evolutionary divergence between Scaevola and its related species
Source: Front Plant Sci. 2025 Apr 24;16:1587750. doi: 10.3389/fpls.2025.1587750 (PMC12058850; doi:10.3389/fpls.2025.1587750)
Supplement: Supplementary file 1 [file DataSheet1.docx]

### Supplementary Table S1. Gene annotation and classification in the chloroplast genomes of two *Scaevola* species

| **Group of genes** | ***S.*** taccada | ***S.*** hainanensis | |
| --- | --- | --- | --- |
| **ATP synthase** | *atpA, atpB, atpE, atpF, atpH, atpI* | | *atpA, atpB, atpE, atpF, atpH, atpI* |
| **NADH** dehydrogenase | *ndhA, ndhA, ndhB, ndhC, ndhD, ndhE, ndhF, ndhF, ndhG, ndhH, ndhH, ndhI, ndhJ, ndhK* | | *ndhA, ndhA, ndhB, ndhC, ndhD, ndhE, ndhF, ndhF, ndhG, ndhH, ndhH, ndhI, ndhJ, ndhK* |
| **photosystem I** | *psaA, psaB, psaC, psaI, psaJ* | | *psaA, psaB, psaC, psaI, psaJ* |
| **p**hotosystem II | *psbA, psbA, psbB, psbC, psbD, psbE, psbF, psbH, psbI, psbJ, psbK, psbL, psbM, psbN, psbT, psbZ, ycf3* | | *psbA, psbA, psbB, psbC, psbD, psbE, psbF, psbH, psbI, psbJ, psbK, psbL, psbM, psbN, psbT, psbZ, ycf3* |
| **cytochrome b/f complex** | *petA, petB, petD, petG, petL, petN* | | *petA, petB, petD, petG, petL, petN* |
| **r**ubisco | *rbcL, rbcL* | | *rbcL, rbcL* |
| **Large** subunit of ribosome | *rpl14, rpl16, rpl2, rpl20, rpl22, rpl22, rpl23, rpl32, rpl33, rpl36* | | *rpl14, rpl16, rpl2, rpl20, rpl22, rpl22, rpl23, rpl32, rpl33, rpl36* |
| **DNA dependent RNA polymerase** | *rpoA, rpoB, rpoC1, rpoC2* | | *rpoA, rpoB, rpoC1, rpoC2* |
| **Small subunit** of ribosome | *rps11, rps12, rps14, rps15, rps15, rps16, rps19, rps2, rps3, rps4, rps7, rps8* | | *rps11, rps12, rps14, rps15, rps15, rps16, rps19, rps2, rps3, rps4, rps7, rps8* |
| **Acetyl-CoA-carboxylase** | *accD, accD* | | *accD, accD* |
| **c-type cytochrome** synthesis gene | *ccsA* | | *ccsA* |
| **Envelop membrane** protein | *cemA* | | *cemA* |
| **Protease** | *clpP* | | *clpP* |
| **Translational initiation factor** | *infA* | | *infA* |
| **Maturase** | *matK*, matK | | *matK*, matK |
| **Conserved open reading** frames | *ycf1, ycf1, ycf2, ycf4* | | *ycf2, ycf4* |

### Supplementary Table S2. Gene annotation and classification in the mitochondrial genomes of two *Scaevola* species

| **Group of genes** | ***S. taccada*** | ***S. hainanensis*** |
| --- | --- | --- |
| **ATP synthase** | *atp4, atp6, atp8* | *atp4, atp6, atp8* |
| **NADH dehydrogenase** | *nad1, nad2, nad3, nad4, nad4L, nad5, nad6, nad7, nad9* | *nad1, nad2, nad3, nad4, nad4L, nad5, nad6, nad7, nad9* |
| **Cytochrome c biogenesis** | *cob* | *cob* |
| **Ubiquinol-cytochrome c reductase** | *ccmB, ccmC, ccmFC, ccmFN* | *ccmB, ccmC, ccmFC, ccmFN* |
| **Cytochrome c oxidase** | *cox1, cox2, cox3* | *cox1, cox2, cox3* |
| **Maturases** | *matR* | *matR* |
| **Transport membrane protein** | *mttB* | *mttB* |
| **Large subunit of ribosome** | *rpl5* | *rpl5* |
| **Small subunit of ribosome** | *rps13* | *rps13* |
| **Ribosome RNA** | *rrn5, rrn18, rrn26* | *rrn5, rrn18, rrn26* |
| **Transfer RNA** | *trnC-GCA, trnD-GUC, trnE-UUC, trnF-GAA, trnG-GCC, trnH-GUG, trnK-UUU, trnM-CAU (×3), trnN-GUU(×2), trnP-UGG(×2), trnQ-UUG, trnW-CCA, trnY-GUA* | *trnC-GCA, trnD-GUC, trnE-UUC, trnF-GAA, trnG-GCC, trnH-GUG, trnK-UUU, trnM-CAU(×3), trnN-GUU(×2), trnP-UGG, trnQ-UUG, trnW-CCA(×2), trnY-GUA* |

**Supplementary Table S3. Relative synonymous codon usage and codon numbers of protein coding genes in the chloroplast genomes of two *Scaevola* species.**

|  |  | ***S. taccada*** | | ***S. hainanensis*** | |  |  | ***S. taccada*** | | ***S. hainanensis*** | |
| --- | --- | --- | --- | --- | --- | --- | --- | --- | --- | --- | --- |
| **AA** | **codon** | **No.** | **RSCU** | **No.** | **RSCU** | **AA** | **codon** | **No.** | **RSCU** | **No.** | **RSCU** |
| **stop codon** | UAA | 55 | 1.88 | 54 | 1.88 | **Leu** | CUA | 413 | 0.93 | 456 | 0.99 |
|  | UAG | 23 | 0.78 | 20 | 0.7 |  | CUC | 152 | 0.34 | 147 | 0.32 |
|  | UGA | 10 | 0.34 | 12 | 0.42 |  | CUG | 168 | 0.38 | 154 | 0.34 |
| **Ala** | GCA | 399 | 1.08 | 400 | 1.08 |  | CUU | 551 | 1.24 | 563 | 1.23 |
|  | GCC | 220 | 0.6 | 225 | 0.61 |  | UUA | 836 | 1.88 | 893 | 1.94 |
|  | GCG | 180 | 0.49 | 182 | 0.49 |  | UUG | 555 | 1.24 | 544 | 1.18 |
|  | GCU | 678 | 1.84 | 673 | 1.82 | **Lys** | AAA | 928 | 1.49 | 1176 | 1.5 |
| **Arg** | AGA | 415 | 1.78 | 469 | 1.92 |  | AAG | 320 | 0.51 | 389 | 0.5 |
|  | AGG | 143 | 0.61 | 154 | 0.63 | **Met** | AUG | 565 | 1.00 | 585 | 1 |
|  | CGA | 303 | 1.3 | 304 | 1.25 | **Phe** | UUC | 488 | 0.68 | 520 | 0.68 |
|  | CGC | 117 | 0.5 | 113 | 0.46 |  | UUU | 942 | 1.32 | 1017 | 1.32 |
|  | CGG | 100 | 0.43 | 106 | 0.43 | **Pro** | CCA | 262 | 1.05 | 266 | 1.04 |
|  | CGU | 320 | 1.37 | 319 | 1.31 |  | CCC | 189 | 0.76 | 196 | 0.77 |
| **Asn** | AAC | 250 | 0.49 | 275 | 0.47 |  | CCG | 127 | 0.51 | 132 | 0.52 |
|  | AAU | 777 | 1.51 | 905 | 1.53 |  | CCU | 416 | 1.67 | 427 | 1.67 |
| **Asp** | GAC | 181 | 0.37 | 170 | 0.34 | **Ser** | AGC | 146 | 0.49 | 143 | 0.46 |
|  | GAU | 788 | 1.63 | 818 | 1.66 |  | AGU | 377 | 1.27 | 375 | 1.2 |
| **Cys** | UGC | 77 | 0.57 | 84 | 0.6 |  | UCA | 315 | 1.06 | 346 | 1.11 |
|  | UGU | 194 | 1.43 | 197 | 1.4 |  | UCC | 277 | 0.93 | 289 | 0.93 |
| **Gln** | CAA | 655 | 1.57 | 687 | 1.56 |  | UCG | 155 | 0.52 | 159 | 0.51 |
|  | CAG | 178 | 0.43 | 194 | 0.44 |  | UCU | 514 | 1.73 | 559 | 1.79 |
| **Glu** | GAA | 991 | 1.50 | 1121 | 1.49 | **Thr** | ACA | 329 | 1.15 | 365 | 1.12 |
|  | GAG | 326 | 0.50 | 379 | 0.51 |  | ACC | 186 | 0.65 | 203 | 0.62 |
| **Gly** | GGA | 593 | 1.42 | 624 | 1.47 |  | ACG | 113 | 0.40 | 154 | 0.47 |
|  | GGC | 251 | 0.60 | 243 | 0.57 |  | ACU | 512 | 1.80 | 578 | 1.78 |
|  | GGG | 290 | 0.69 | 277 | 0.65 | **Trp** | UGG | 410 | 1.00 | 430 | 1 |
|  | GGU | 538 | 1.29 | 555 | 1.31 | **Tyr** | UAC | 164 | 0.36 | 186 | 0.39 |
| **His** | CAC | 125 | 0.47 | 128 | 0.45 |  | UAU | 748 | 1.64 | 780 | 1.61 |
|  | CAU | 410 | 1.53 | 445 | 1.55 | **Val** | GUA | 474 | 1.49 | 480 | 1.45 |
| **Ile** | AUA | 627 | 0.92 | 745 | 0.99 |  | GUC | 188 | 0.59 | 197 | 0.6 |
|  | AUC | 394 | 0.58 | 426 | 0.57 |  | GUG | 146 | 0.46 | 154 | 0.47 |
|  | AUU | 1014 | 1.49 | 1082 | 1.44 |  | GUU | 462 | 1.46 | 493 | 1.49 |

**Supplementary Table S4. Relative synonymous codon usage and codon numbers of protein coding genes in the mitochondrial genomes of two *Scaevola* species.**

|  |  | ***S. taccada*** | | ***S. hainanensis*** | |  |  | 1. ***taccada*** | | ***S.hainanensis*** | |
| --- | --- | --- | --- | --- | --- | --- | --- | --- | --- | --- | --- |
| **AA** | **codon** | **No.** | **RSCU** | **No.** | **RSCU** | **AA** | **codon** | **No.** | **RSCU** | **No.** | **RSCU** |
| **stop codon** | UAA | 13 | 1.62 | 12 | 1.5 | **Leu** | CUA | 141 | 0.91 | 144 | 0.88 |
|  | UAG | 3 | 0.38 | 3 | 0.38 |  | CUC | 99 | 0.64 | 107 | 0.65 |
|  | UGA | 8 | 1 | 9 | 1.12 |  | CUG | 76 | 0.49 | 79 | 0.48 |
| **Ala** | GCA | 120 | 0.95 | 126 | 0.98 |  | CUU | 218 | 1.41 | 237 | 1.45 |
|  | GCC | 117 | 0.93 | 116 | 0.9 |  | UUA | 228 | 1.47 | 234 | 1.43 |
|  | GCG | 63 | 0.5 | 65 | 0.5 |  | UUG | 167 | 1.08 | 180 | 1.1 |
|  | GCU | 205 | 1.62 | 208 | 1.62 | **Lys** | AAA | 131 | 1.05 | 138 | 1.05 |
| **Arg** | AGA | 92 | 1.25 | 90 | 1.18 |  | AAG | 119 | 0.95 | 124 | 0.95 |
|  | AGG | 65 | 0.88 | 78 | 1.02 | **Met** | AUG | 211 | 1 | 215 | 1 |
|  | CGA | 95 | 1.29 | 98 | 1.28 | **Phe** | UUC | 237 | 0.88 | 247 | 0.89 |
|  | CGC | 37 | 0.5 | 38 | 0.5 |  | UUU | 304 | 1.12 | 308 | 1.11 |
|  | CGG | 54 | 0.73 | 55 | 0.72 | **Pro** | CCA | 119 | 1.13 | 121 | 1.11 |
|  | CGU | 98 | 1.33 | 99 | 1.3 |  | CCC | 84 | 0.8 | 89 | 0.82 |
| **Asn** | AAC | 63 | 0.58 | 67 | 0.58 |  | CCG | 54 | 0.51 | 58 | 0.53 |
|  | AAU | 155 | 1.42 | 166 | 1.42 |  | CCU | 163 | 1.55 | 168 | 1.54 |
| **Asp** | GAC | 83 | 0.68 | 90 | 0.71 | **Ser** | AGC | 84 | 0.71 | 90 | 0.73 |
|  | GAU | 160 | 1.32 | 162 | 1.29 |  | AGU | 126 | 1.06 | 132 | 1.07 |
| **Cys** | UGC | 52 | 0.98 | 50 | 0.9 |  | UCA | 130 | 1.09 | 134 | 1.08 |
|  | UGU | 54 | 1.02 | 61 | 1.1 |  | UCC | 113 | 0.95 | 120 | 0.97 |
| **Gln** | CAA | 153 | 1.51 | 162 | 1.5 |  | UCG | 85 | 0.71 | 87 | 0.7 |
|  | CAG | 49 | 0.49 | 54 | 0.5 |  | UCU | 176 | 1.48 | 180 | 1.45 |
| **Glu** | GAA | 198 | 1.37 | 212 | 1.34 | **Thr** | ACA | 85 | 0.85 | 90 | 0.87 |
|  | GAG | 92 | 0.63 | 104 | 0.66 |  | ACC | 107 | 1.08 | 114 | 1.1 |
| **Gly** | GGA | 181 | 1.39 | 186 | 1.39 |  | ACG | 60 | 0.6 | 62 | 0.6 |
|  | GGC | 74 | 0.57 | 77 | 0.58 |  | ACU | 146 | 1.47 | 147 | 1.42 |
|  | GGG | 96 | 0.74 | 101 | 0.76 | **Trp** | UGG | 136 | 1 | 140 | 1 |
|  | GGU | 169 | 1.3 | 171 | 1.28 | **Tyr** | UAC | 63 | 0.5 | 63 | 0.48 |
| **His** | CAC | 46 | 0.48 | 49 | 0.49 |  | UAU | 188 | 1.5 | 199 | 1.52 |
|  | CAU | 147 | 1.52 | 149 | 1.51 | **Val** | GUA | 137 | 1.15 | 140 | 1.14 |
| **Ile** | AUA | 163 | 0.79 | 174 | 0.83 |  | GUC | 101 | 0.85 | 108 | 0.88 |
|  | AUC | 194 | 0.94 | 201 | 0.96 |  | GUG | 103 | 0.86 | 104 | 0.84 |
|  | AUU | 259 | 1.26 | 255 | 1.21 |  | GUU | 136 | 1.14 | 141 | 1.14 |

**Supplementary Table S5. The dispersed repeats (A) and SSR (B) in the mitochondrial genomes of two *Scaevola* species.**

| **A.** | |  | | | | | |  | | | | | |
| --- | --- | --- | --- | --- | --- | --- | --- | --- | --- | --- | --- | --- | --- |
|  | | ***S. taccada*** | | | | | | | ***S. hainanensis*** | | | | |
|  |  | No. | | Min length | | Max length | | | No. | Min length | | Max length | |
| Forward repeats | | 66 | | 30 | | 137 | | | 42 | 30 | | 102 | |
| palindromic repeats | | 80 | | 30 | | 3871 | | | 42 | 30 | | 169 | |
| reverse repeats | | 1 | | 33 | | 33 | | | 1 | 33 | | 33 | |
| **B.** |  | |  | |  | |  | | |  |  | |  |
| **SSR type** | | ***S. taccada*** | | | | | | ***S. hainanensis*** | | | | | |
| P1 | | 11 | | | | | | 4 | | | | | |
| P2 | | 2 | | | | | | 2 | | | | | |
| P3 | | 2 | | | | | | 1 | | | | | |
| P4 | | 32 | | | | | | 25 | | | | | |
| P5 | | 6 | | | | | | 7 | | | | | |
| P6 | | 0 | | | | | | 1 | | | | | |
| All | | 53 | | | | | | 40 | | | | | |
