# Supplementary material for: Organelle genomes of two Scaevola species, S. taccada and S. hainanensis, provide new insights into evolutionary divergence between Scaevola and its related species
Source: Front Plant Sci. 2025 Apr 24;16:1587750. doi: 10.3389/fpls.2025.1587750 (PMC12058850; doi:10.3389/fpls.2025.1587750)
Supplement: Supplementary file 2 [file DataSheet2.docx]

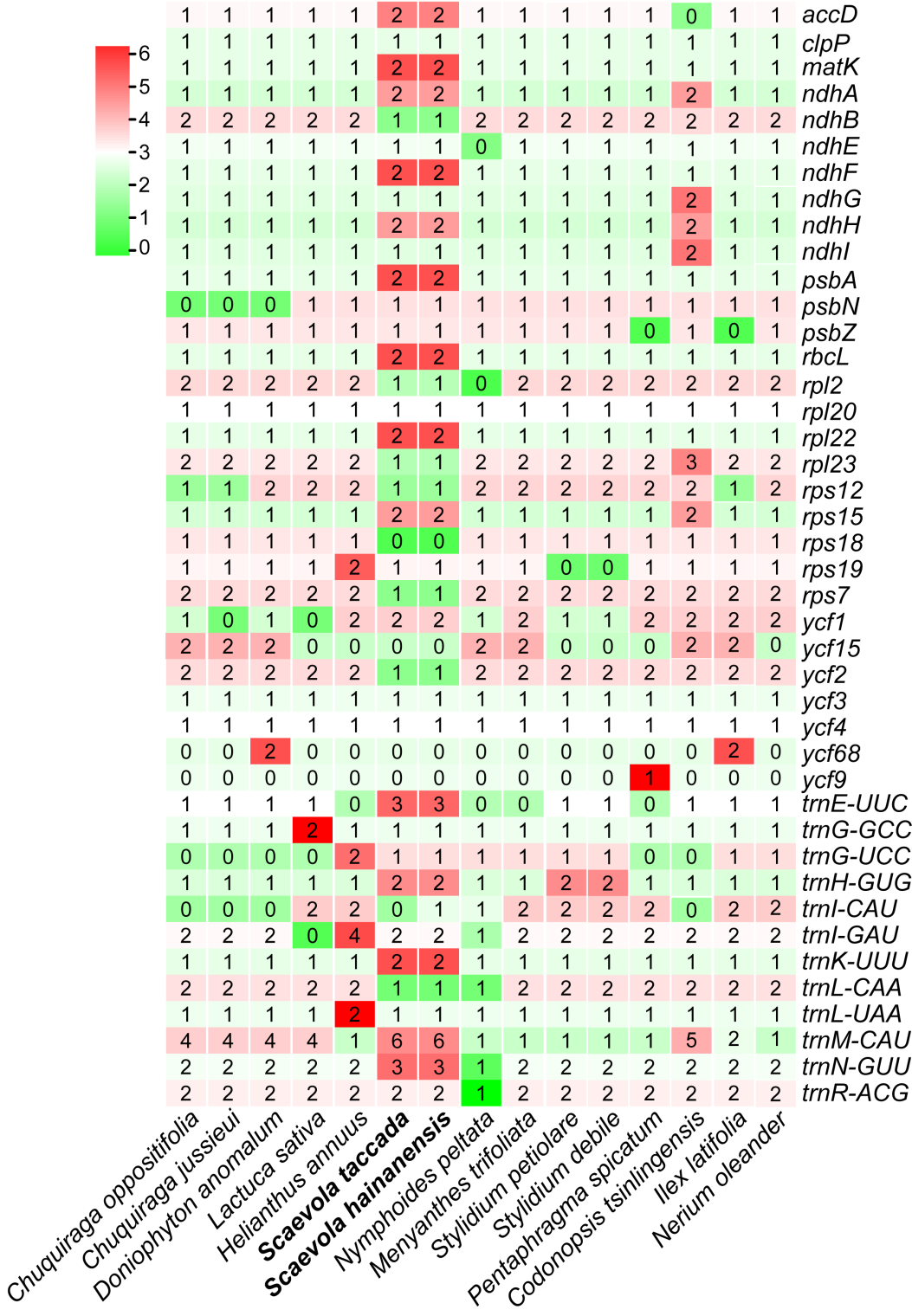


**Supplementary Figure S1. The copy number of genes varies among the 15 chloroplast genomes.**


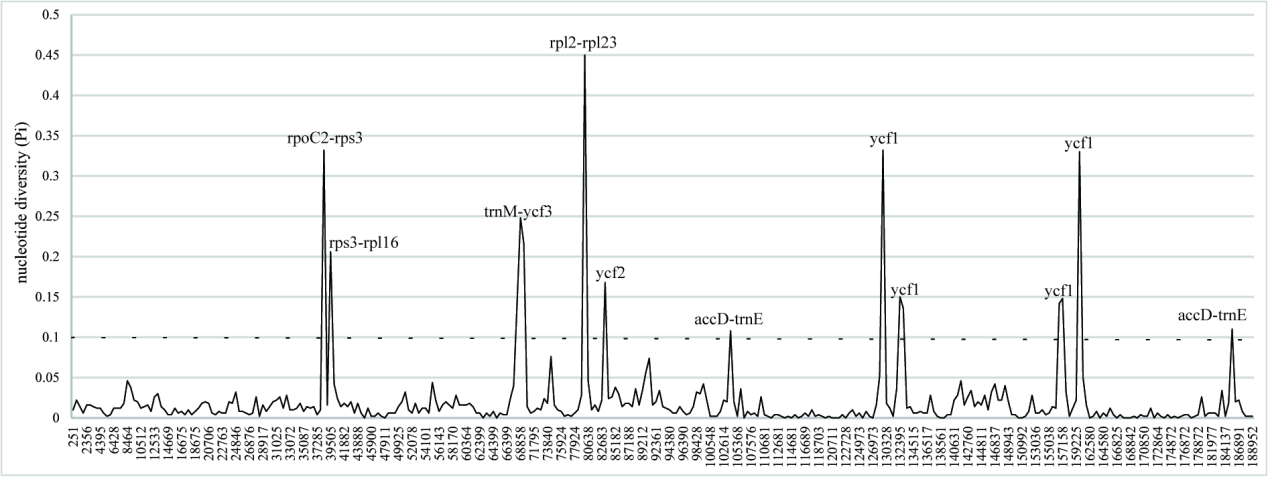


**Supplementary Figure S2. Comparison of the Pi value in two** ***Scaevola* species.**


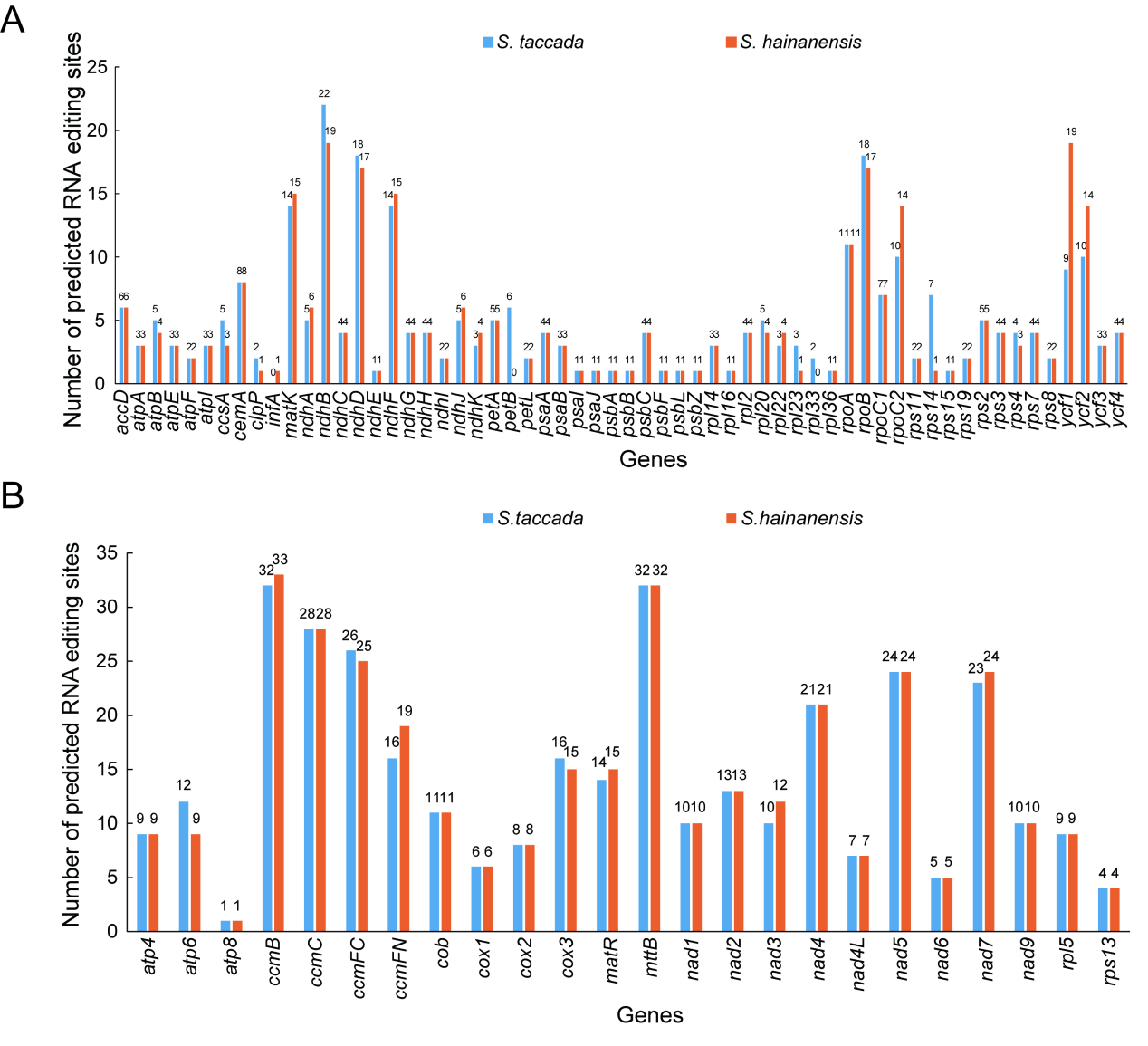


### **Supplementary Figure S3. Prediction of RNA editing sites in chloroplast (A) and mitochondrial (B) genes of two *Scaevola* species.**


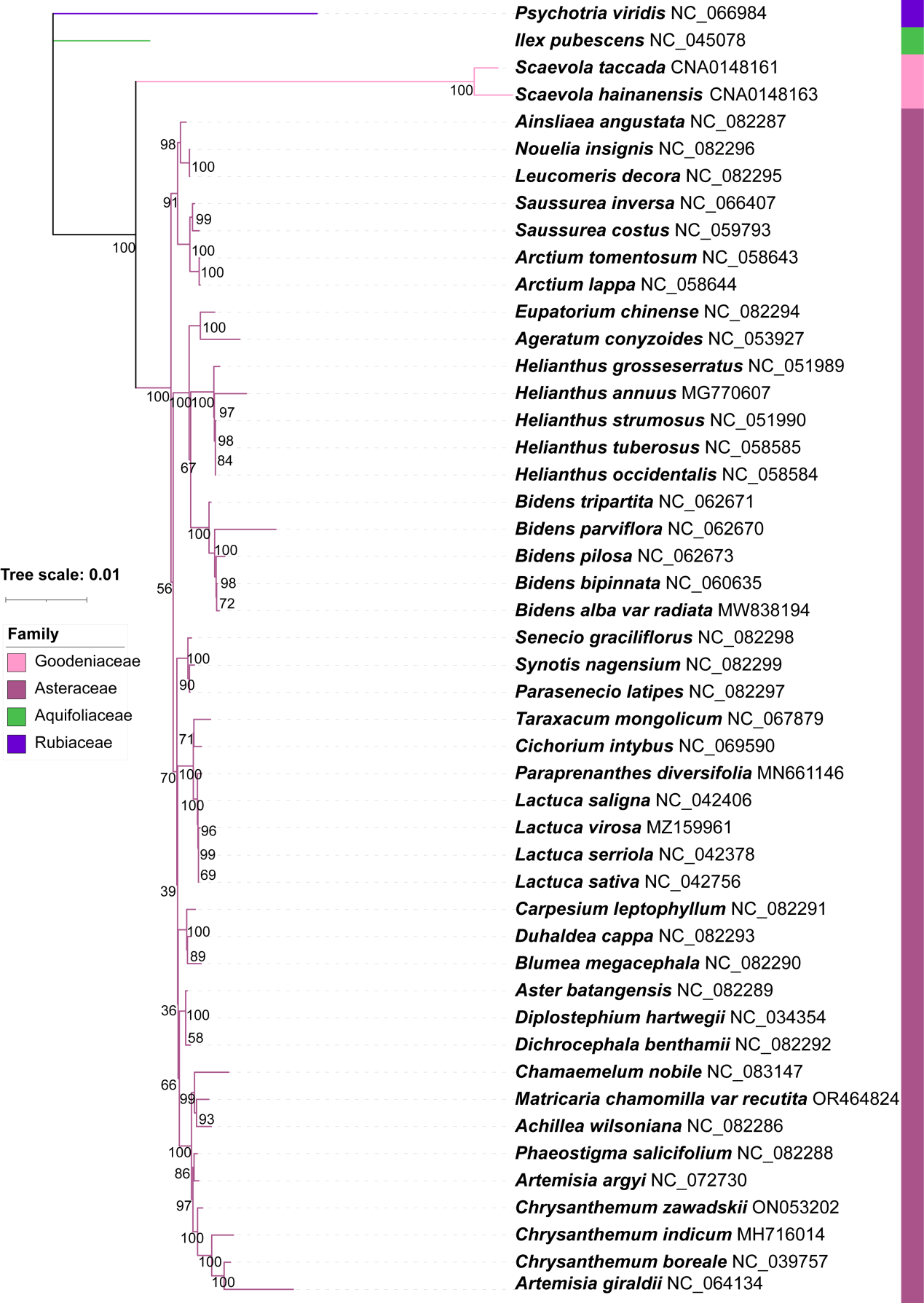


**Supplementary Figure S4. Phylogenetic analysis of 48 Asterales plants using mitochondrial genome sequences. The tree was constructed using 18 conserved protein codon genes from those species via IQtree program. The bootstrap score obtained using 1000 replicates. *Ilex pubescens* and *Psychontria viridis* were used as outgroups.**


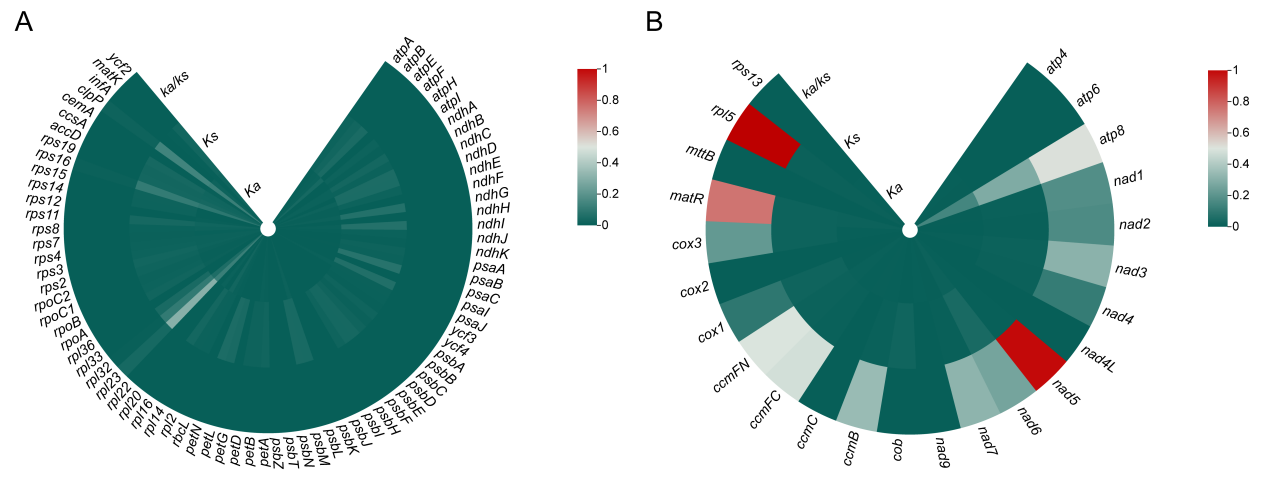


**Supplementary Figure S5. Selective pressure analysis of chloroplast (A) and mitochondrial (B) coding genes of two *Scaevola* species.** Synonymous (*Ks*) and nonsynonymous (*Ka*) substitution rates for organellar protein coding genes were calculated by DNaSP5. Heat maps of *Ka*, *Ks* and *Ka/Ks* ratios were visualized by Chiplot online tools (https://www.chiplot.online/). Gene names are indicated outside the circles. Note that all chloroplast coding genes exhibit *Ka/Ks* values less than 0.3, whereas most of mitochondrial coding genes exhibit *Ka/Ks* over 0.4, with *Ka/Ks* ratios of *ndh5* and *rpl5* over 1, indicating potential positive selection for these two genes.


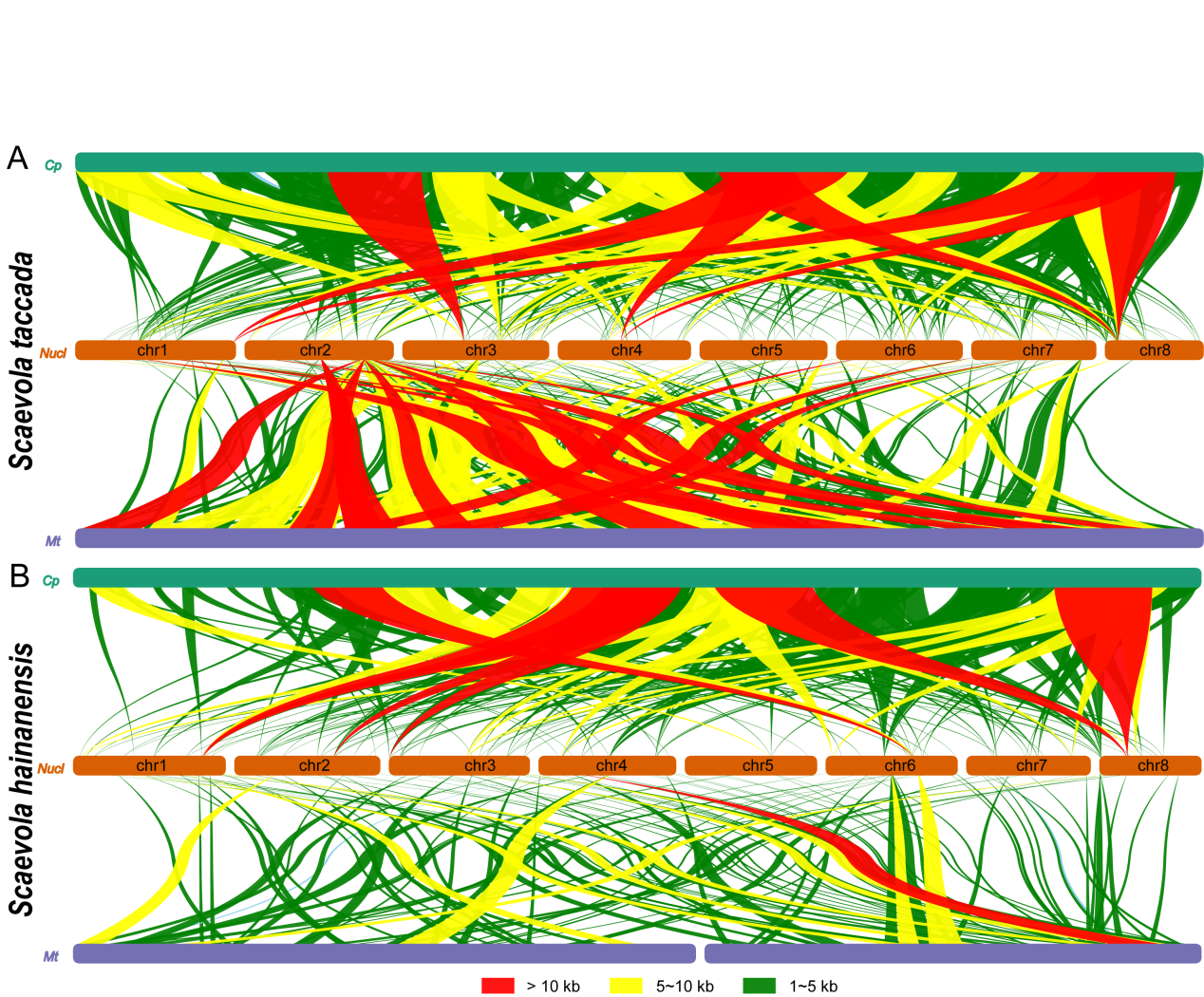


**Supplementary Figure S6. Schematic representations of MTPT and NUMT of two *Scaevola* species.**
